# The role of immune dysregulation of innate and adaptive immune cells in the prodromal and clinical stages of Parkinson’s disease

**DOI:** 10.1002/alz.085589

**Published:** 2025-01-09

**Authors:** Julian R Mark, Rebecca L Wallings, Hannah Staley, Malu Gámez Tansey

**Affiliations:** ^1^ University of Florida College of Medicine, Gainesville, FL USA

## Abstract

**Background:**

Peripheral blood mononuclear cells (PBMCs) were obtained from patients across different stages of Parkinson’s disease (PD) progression and stimulated *ex vivo* to develop biomarkers for predicting PD progression.

**Method:**

PBMCs obtained at one time‐point from patients with moderate stage PD (>5 years after diagnosis) (n = 30), early stage PD (<5 years after diagnosis) (n = 27), prodromal PD (rapid‐eye‐movement sleep behavior disorder patients) (n = 14), and healthy controls (HCs) (n = 9) were isolated from whole blood and cryopreserved. Samples were thawed, then pan‐monocytes and T‐cell populations were isolated from PBMCs and subjected to treatment with vehicle or IFN‐γ. An additional treatment condition with CD3/28 magnetic beads was included for T‐cells. Cultured media was kept for evaluation of cell‐type specific cytokine release, and live cells were analyzed with flow cytometry for markers of immune activation, mitochondrial and lysosomal function, and LRRK2 expression and activity.

**Result:**

In classical, intermediate, and non‐classical monocytes from HCs, expression of pRab10, LRRK2, and MHC‐II increases after treatment with *ex vivo* IFN‐γ stimulation. In CD4+ and CD8+ T‐cells from HCs, IFN‐γ and CD3/28 bead treatment caused increased expression of pRab10. CD3/28 magnetic beads, but not IFN‐γ, caused increased expression of CD137 and LRRK2, while also leading to reduced levels of acidified lysosomes. Studies are currently underway to analyze cells from PD and prodromal patients and evaluate cell‐type specific cytokine release in the conditioned media.

**Conclusion:**

We have applied a validated *ex vivo* stimulation protocol using PBMCs from PD patients across different stages of disease to measure immune cell activation and cell‐type specific cytokine release. If our hypothesis is correct, we expect that immune cell subsets from PD patients will display a signature of dysregulated immune response to stimulation. Furthermore, we expect that prodromal PD patients will exhibit dysregulated peripheral immune responses compared to controls, including altered mitochondrial and lysosomal function and cytokine release.

